# Spontaneous Early Resolution of an Iatrogenic Type A Aortic Dissection Following Coronary Angiography

**DOI:** 10.1055/s-0039-1683956

**Published:** 2019-04-24

**Authors:** Yavuzer Koza, Uğur Kaya, Hakan Taş, Enise Armagan Koza

**Affiliations:** 1 Department of Cardiology, Ataturk University Faculty of Medicine, Erzurum, Turkey; 2 Department of Cardiovascular Surgery, Ataturk University Faculty of Medicine, Erzurum, Turkey; 3 Department of Anesthesiology, Erzurum Regional Training and Research Hospital, Erzurum, Turkey

**Keywords:** iatrogenic aortic dissection, coronary angiography, aorta, medical therapy

## Abstract

A 74-year-old man was admitted with the diagnosis of non–ST-elevation myocardial infarction. During right coronary angiography, a coronary artery dissection extending into the proximal ascending aorta was noticed without hemodynamic compromise. Immediate computed tomography angiography showed no evidence of dissection in the ascending aorta. The patient remained hemodynamically stable with medical therapy alone. This case report highlights the importance of medical therapy in patients with uncomplicated iatrogenic aortic dissection.

## Introduction

Aortocoronary dissections occur most commonly during retrograde percutaneous coronary intervention of chronic total occlusions (CTOs) due to more aggressive and extensive osteal atherosclerosis. Indeed, the most common cause of iatrogenic aortic dissections (IADs) is guiding catheter tip trauma. 1 Iatrogenic dissections of the coronary artery and ascending aorta are very rare complications of diagnostic coronary angiography with an incidence of 0.02%. 2 Here, we report the uneventful clinical outcome with conservative management of a patient with catheter-induced acute aortic dissection originating from the right coronary artery (RCA).

## Case Presentation

**Fig. 1:**
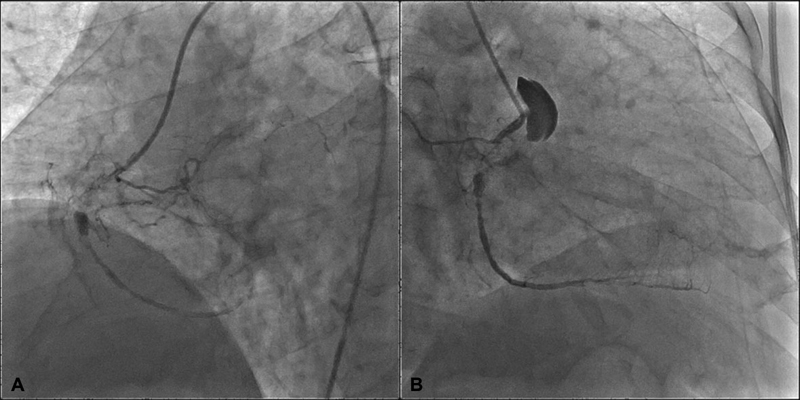
**(A)** Origin of the dissection at the proximal right coronary artery. **(B)** Right anterior oblique (RAO) view showing the extension of the dissection to ascending aorta.

**Fig. 2:**
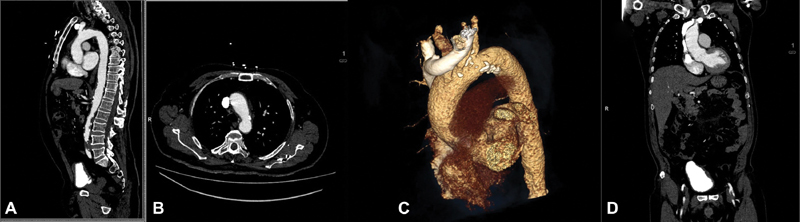
Transverse **(A)** , axial **(B)** , axial volume rendered **(C)** , and sagittal **(D)** Computed tomographic images immediately after coronary angiography showing no evidence of dissection in the ascending aorta.

**Fig. 3:**
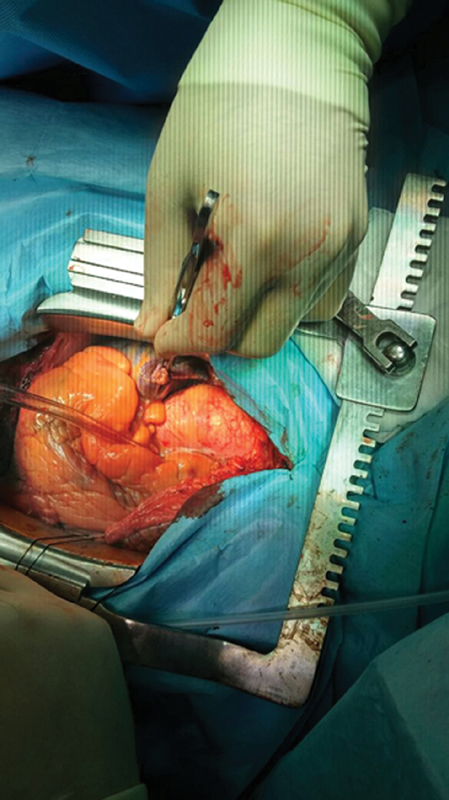
Intraoperative image of the ascending aorta.

A 74-year-old man was hospitalized with the diagnosis of non–ST-elevation myocardial infarction. Diagnostic coronary angiography showed 100% stenosis of left anterior descending (LAD) artery with retrograde flow to the RCA and 80% stenosis in obtuse marginal branch of the left circumflex artery. During the right coronary angiogram in right anterior oblique position, to confirm RCA occlusion, a coronary artery dissection extending into the proximal ascending aorta was noticed without hemodynamic compromise. ( Fig. 1A , B ). Transthoracic echocardiography demonstrated no pericardial effusion. Immediate computed tomographic angiography showed no evidence of dissection in the ascending aorta ( Fig. 2A–D ). The initial and subsequent echocardiogram examinations showed no pericardial effusion or dissection flap. Because the patient was stable with an intact aortic valve and aorta, we decided to pursue a conservative management strategy. Coronary artery bypass surgery was planned for his coronary lesions. A saphenous vein graft to the obtuse marginal branch of the circumflex artery and a left internal thoracic artery to the LAD coronary artery were performed. There was no evidence of the dissection in the aorta ( Fig. 3 ). The patient tolerated the surgery well and was discharged 10 days later.

## Discussion

Clinical manifestations of IADs may vary from an asymptomatic angiographic finding to a complete hemodynamic collapse owing to the closure of the coronary ostium. 2 3 Occurrence of IAD can be prevented by avoiding aggressive manipulations or deep engagement of catheters and maintaining a steady tension on the guiding catheter while the angioplasty balloon is withdrawn. 4 During coronary angiography, the pressure waveform should be monitored to prevent contrast injection in the presence of dampening. If coronary dissection occurs during a diagnostic angiography, exchanging to a guiding catheter with good coaxial alignment should be considered. More importantly, it is essential to stop subsequent contrast injections that could lead to extension and enlargement of the dissection.

In this case, an initial limited iatrogenic dissection of the proximal RCA and subsequent forceful contrast medium injection were the most likely causes of this dissection.

Iatrogenic coronary artery dissections are classified into three forms by Dunning et al. 5 Coronary stenting is the recommended management strategy for classes I (cusp only) and II (up the aortic wall < 4 cm) dissections. Class III (up the aortic wall > 4 cm) dissections should be preferably treated surgically, because they involve aortic valve cusps and extend up the aorta. 5

The appropriate therapeutic strategy for iatrogenic dissections remains controversial being generally based on the type of dissection and the hemodynamic stability of the patient. Some authors have proposed that in all the cases in which there is dissection into the coronary sinus, the ostium should be sealed immediately by ostial stenting. 6 Others have proposed that surgical intervention should be considered if the dissection extends to 4 cm or beyond into the aorta. 4 Spontaneous regression of IADs has also been reported in case of limited IAD, probably due to spontaneous sealing and stagnation of blood flow in the false lumen. 7 Park et al 8 reported a good outcome of a CTO patient who suffered AD involving the entire ascending aorta, successfully managed using a conservative strategy. Indeed, a more recent case was reported by Wykrzykowska et al, 9 underscoring the strategy consisting of patching up the coronary problem, and “wait and see” in close intervals using the computed tomographic scan or transesophageal echocardiography, if the dissected aortic wall segment will heal. However, it is not possible to know which patient might be conservatively managed with “wait and see” in such a dangerous complication. We think that the extent of dissection is a significant prognostic factor, and in cases such as ours, a relative intact aorta without heavy calcification is the most important factor in spontaneous healing. This case may be useful for cardiologist to avoid RCA stenting or at least to think about the best approach in the case without ischemic and hemodynamic signs.

This case suggests that the selected patients with uncomplicated ascending IADs can be successfully managed by medical therapy alone.
